# Early initiation of renal replacement treatment in patients with acute kidney injury: A systematic review and meta-analysis

**DOI:** 10.1097/MD.0000000000005434

**Published:** 2016-11-18

**Authors:** Hongwei Wang, Liwei Li, Qinjun Chu, Yong Wang, Zhisong Li, Wei Zhang, Lanlan Li, Long He, Yanqiu Ai

**Affiliations:** Department of Anesthesiology, The First Affiliated Hospital of Zhengzhou University, Zhengzhou, Henan, PR China.

**Keywords:** acute kidney injury, renal replacement therapy, time factors

## Abstract

**Background:**

Acute kidney injury (AKI) is associated with a substantially increased risk of mortality for many hospitalized patients. It has been suggested that early initiation of renal replacement treatment has a favorable outcome in critically ill patients complicated with AKI. However, results of studies evaluating the effect of early initiation strategy of renal replacement treatment on AKI have been controversial and contradictory. The aim of this meta-analysis is to examine the effect of early initiation of renal replacement treatment on patients with AKI.

**Methods:**

The authors searched relevant studies in PubMed, EMBASE, and the Cochrane Library through August 2016. We searched for all eligible randomized controlled trials with regard to the role of early initiation of renal replacement treatment in mortality among patients with AKI. We extracted the following information from each study: mortality, length of stay in intensive care unit (ICU), and length of stay in hospital. Random and fixed effect models were used for pooling data.

**Results:**

Twelve trials including 1756 patients were included. The results of this meta-analysis showed that there was no significant difference between the mortality of early and delayed strategy for the initiation of renal replacement treatment using the random effect model (odds ratio = 0.78; 95% confidence interval [CI], 0.52–1.19; *P* = 0.25), with wild heterogeneity (chi^2^ = 33.50; *I*^2^ = 67%). Analyses from subgroup sepsis and postsurgery came to similar results. In addition, compared with delayed initiation strategy, early initiation showed no significant advantage in length of stay in ICU (mean difference = −0.80; 95% CI, −2.59 to 0.99; *P* = 0.56) and length of stay in hospital (mean difference = −7.69; 95% CI, −16.14 to 0.76; *P* = 0.07).

**Conclusion:**

According to the results from present meta-analysis, early initiation of renal replacement treatment showed no survival benefits in patients with AKI. To achieve optimal timing of renal replacement treatment, further large multicenter randomized trials, with widely accepted and standardized definition of early initiation, are still needed.

## 1 Introduction

Acute kidney injury (AKI) is a well recognized multidisciplinary complication of critical illness, which can lead to abrupt loss of kidney function and is associated with a substantially increased risk of mortality for many hospitalized patients.^[1–4]^ Renal replacement therapy (RRT) is the cornerstone of the management of patients with severe kidney injury and benefits the recovery of renal function.^[4]^ RRT in patients with AKI could prevent uremia and other adverse complications of renal failure. It has been an important part of treatment and is considered an established treatment modality for patients with AKI.^[5]^

Wide variations, such as timing of initiation, modalities, and dosing, may affect clinical outcomes, particularly survival, although few studies have directly examined these issues.^[6]^ RRT initiation in critically ill patients is complex and conditional on numerous factors. Early initiation means to timely initiate RRT before condition worsened to the degree where the patients become relatively resistant to therapy.^[7,8]^ It has been suggested that early initiation of RRT has a favorable outcome in critically ill patients complicated with AKI.^[9–11]^ However, some other evidence suggests an absence of benefits from an early strategy compared with a delayed one for the initiation of RRT in patients with AKI.^[4,12]^ In addition, the definitions of early initiation in these studies vary from each other. As a result, the studies concerning early initiation of RRT in patients with AKI were inconsistent, and the results remained controversial and contradictory.

Even if some evidence regarding initiation of RRT has been produced, RRT optimal timing still remains uncertain. Recently, several high-quality randomized trials involving the timing of RRT initiation in patients with AKI have been reported. In this study, we conducted a meta-analysis, which extracted results from recently published randomized controlled trials (RCTs) to investigate whether early initiation of RRT in critically ill patients with AKI improves patient survival.

## 2 Methods

### 2.1 Search strategy

The systematic review was performed in accordance with Preferred Reporting Items for Systematic reviews and Meta-Analyses guidelines.^[13]^ Ethical approval was not required considering the nature of the study.

We searched MEDLINE, EMBASE, and the Cochrane Library through August 2016 without language restriction. The search terms were acute kidney injury, acute renal failure, renal replacement therapy, dialysis, hemodialysis, hemofiltration, time to treatment, time factors, early, earlier, time, accelerate, late, initiation, start, and randomized controlled trials. We further identified studies by reviewing the reference lists of relevant papers identified and by discussing with experts in the field to identify unpublished data.

### 2.2 Types of outcome measures

The primary outcome was mortality in early and delayed initiation of RRT. Intensive care unit (ICU) and hospital lengths of stay were secondary outcomes. Weighted means were calculated based on the number of patients in each study.

### 2.3 Study selection

The inclusion criteria were as follows: definite description of factors related to timing of initiation of RRT, diagnosis of AKI, RCT/quasi-RCT, and sufficient data available to calculate a risk ratio (RR) or mean difference (MD) with 95% confidence interval (CI). The following exclusion criteria were used: studies without relevant result, study protocols, pediatric patients, and nonhuman studies.

Two investigators (HW and QC) independently reviewed all abstracts; included the full text of each trial independently; and recorded eligibility, quality, and outcomes. Disagreements between the reviewers concerning the decision to include or exclude a study were resolved through discussion. If necessary, the third reviewer (LL) would be consulted. We excluded duplicate reports, RCTs, and experimental design. Conference abstracts were also excluded, unless published as full-text reports in journals.

### 2.4 Quality assessment

Two reviewers (HW and QC) independently performed quality assessment. We assessed the quality of the trails according to randomization, blinding, and withdrawals and dropouts in line with the Jadad scoring system (range from 0 to 5).^[14]^ We judged the trails as low-quality study with 2 or less points and high-quality study with 3 or more points.

### 2.5 Statistical analysis

Before the analysis, we converted data standardly into equivalent units. We calculated, and subsequently pooled in independent meta-analyses, RR with 95% CI for dichotomous outcomes and MD with 95% CI for continuous outcomes. Heterogeneity among pooled studies was evaluated by the Mantel–Haenszel chi-square test. We assessed the degree of interstudy variation according to the *I*^2^.^[15]^ Homogeneity assumption was measured by *P* value. If a *P* value was less than 0.10, it suggested the evidence of statistically significant heterogeneity, and synthesis of each study was performed by the random effects model.

In this study, we evaluated publication bias by Begg test and Egger test. Sensitivity analysis was conducted by sequentially deleting a single study each time in an attempt to identify the potential influence of each study. A 2-tailed *P* value <0.05 was considered a criterion for statistical significance. All analyses were analyzed by Review Manager 5.3 (RevMan, The Cochrane Collaboration, Oxford, United Kingdom) and STATA 12.0 (StataCorp, College Station, TX).

## 3 Result

### 3.1 Study characteristics

**Figure 1:**
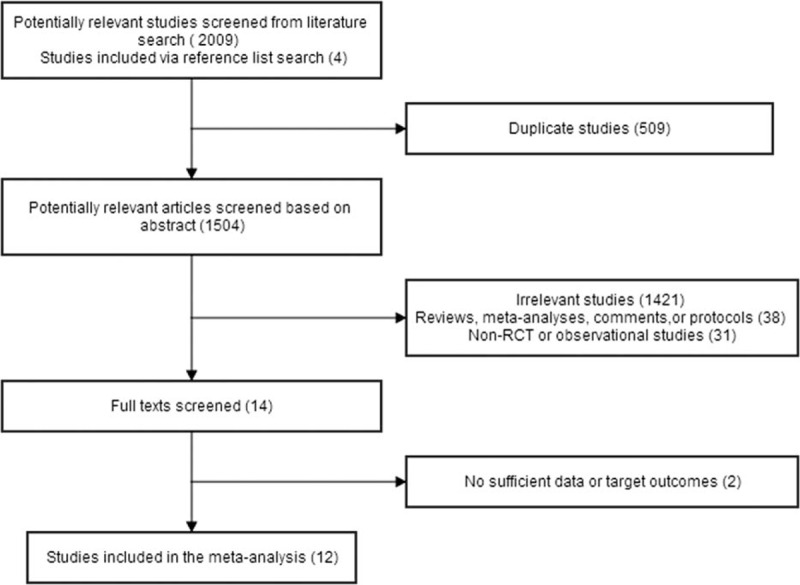
Flowchart of selection process of eligible studies. RCT = randomized controlled trial.

The flowchart of study selection process is presented in Fig. 1. The search strategy identified 2009 studies, and the data were from 12 RCTs comprising 1756 patients (Table 1).^[4,12,16–25]^ One of these trails is conference abstract,^[21]^ which was confirmed to have not been published as full-text report in a peer-reviewed journal. Jadad scores of 4 trails were less than 3.^[16,17,19,21]^ After discussion, we regrettably excluded 2 well designed randomized controlled observational cohort studies.^[26,27]^

**Table 1:**
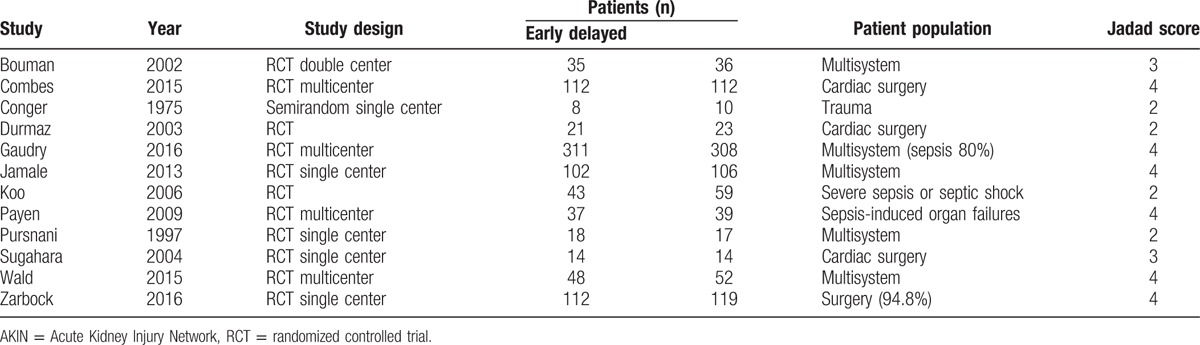
Characteristics of studies included in meta-analysis.

### 3.2 Primary outcome

**Figure 2:**
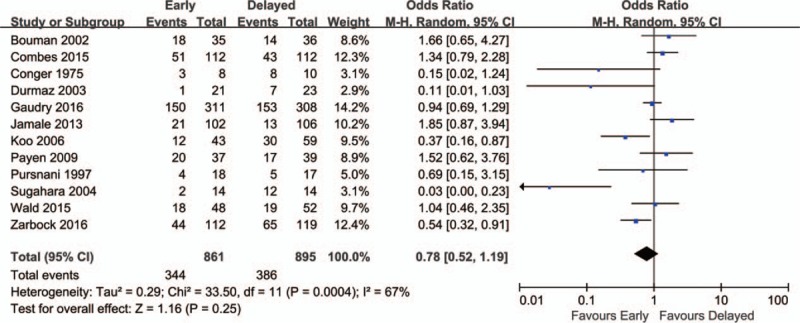
Forest plot for overall mortality.

A total of 12 RCTs including 1756 patients were included, and the overall mortality in patients with AKI was about 41.57% (344/861 in the early group and 386/895 in the delayed group). There was no significant difference between overall mortality of early and delayed strategy for the initiation of RRT using the random effect model (odds ratio [OR] = 0.78; 95% CI, 0.52–1.19; *P* = 0.25), with wild heterogeneity (chi^2^ = 33.50, *I*^2^ = 67%) (Fig. 2). Sensitivity analysis sequentially deleting a single study each time revealed that most individual study was consistent. No significant publication bias was detected, with *P* = 0.193 in Begg test and *P* = 0.155 in Egger test.

**Figure 3:**
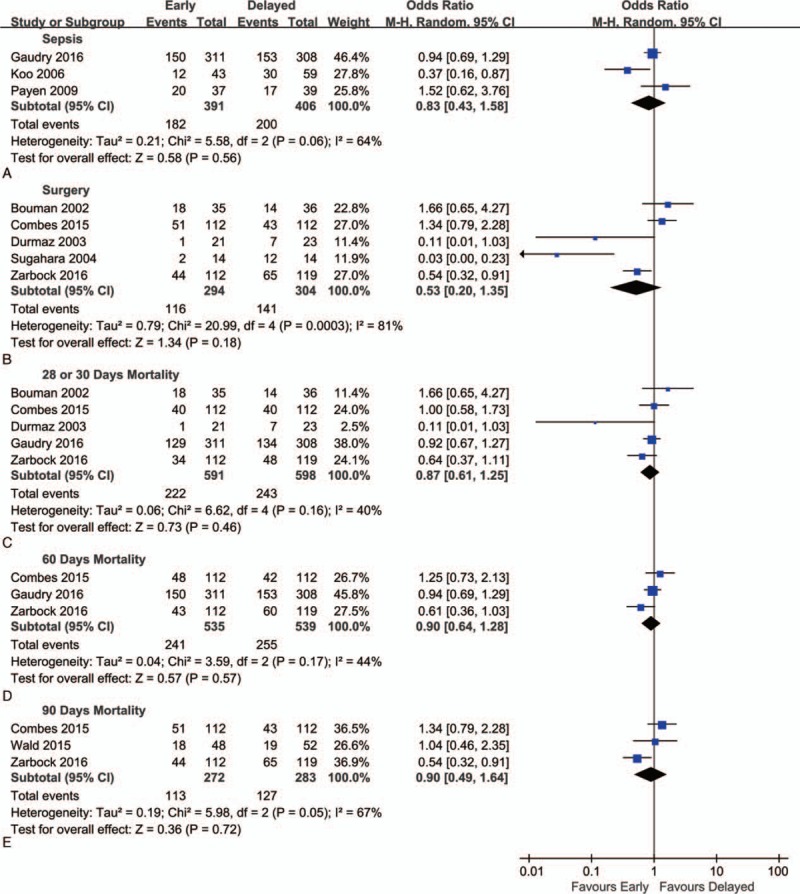
Forest plot for subgroup analyses of mortality. (A) Subgroup analysis for patients with sepsis. (B) Subgroup analysis for patients after surgery. (C–E) Subgroup analysis for mortality at days 30, 60, and 90, respectively.

Subgroup analyses were conducted according to the etiology. Early initiation did not reduce the mortality in subgroup of sepsis (OR = 0.83; 95% CI, 0.43–1.58; *P* = 0.56) (Fig. 3A). Subgroup analysis from patients after surgery also found that early initiation did not lower mortality compared with the delayed strategy for the initiation of RRT (OR = 0.72; 95% CI, 0.31–1.70; *P* = 0.46) (Fig. 3B).

We analyzed mortalities at 3 different time points (day 28 or 30, day 60, and day 90). But early initiation of RRT failed to show any advantage at each time point (Fig. 3C–E).

### 3.3 Secondary outcomes

#### 3.3.1 Effect of early initiation of RRT on length of ICU stay

**Figure 4:**
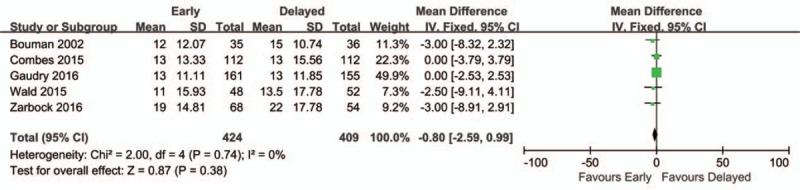
Forest plot for length of stay in intensive care unit.

Five of included studies were analyzed to assess effect of early initiation of RRT on length of ICU stay. There was no statistically significant difference in the overall mortality between 2 groups (mean difference, −0.80; 95% CI, −2.59 to 0.99; *P* = 0.38) with no heterogeneity (chi^2^ = 2.00; *I*^2^ = 0%) (Fig. 4).

#### 3.3.2 Effect of early initiation of RRT on length of hospital stay

**Figure 5:**
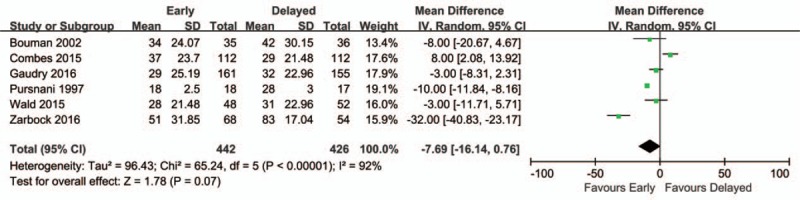
Forest plot for length of stay in hospital.

Available information on the length of hospital stay was analyzed. No statistically significant difference was observed between early and delayed initiation of RRT (mean difference, −7.69; 95% CI, −16.14 to 0.76; *P* = 0.07) (Fig. 5).

## 4 Discussion

The meta-analysis reported detailed analyses of 12 trails comparing early with delayed initiation of RRT on AKI. The results of this meta-analysis showed no significant difference of mortality between an early and a delayed strategy for the initiation of RRT. No significant differences were found in length of stay in ICU and that in hospital.

AKI is a common disease or complication in critically ill patients in ICU. Among patients with AKI requiring RRT, in-hospital mortality rates ranged from 20% to 60.3% when accompanied with nonrenal organ system failure.^[28,29]^ Several studies showed high survival rates and kidney recovery among patients who received early RRT.^[30–32]^ Recently, a single-center trial,^[25]^ comparing early RRT with delayed RRT in patients with AKI trial, reported that early initiation resulted in a 15.4% reduction in 90-day mortality compared with delayed RRT (39.3% vs 53.6%; *P* = 0.03). For the early group, RRT was initiated within 8 hours of diagnosis of stage 2 AKI using the Kidney Disease: Improving Global Outcomes (KDIGO) classification, while delayed RRT was initiated within 12 hours of stage 3 AKI. Early RRT also showed shorter hospital stay and reduction in selected plasma proinflammatory mediators. There were no differences in organ dysfunction scores or dialysis dependence beyond 90 days. However, in this research, the vast majority (94.8%) of patients were from surgical ICU.

Other studies showed no significant survival or renal function benefit compared with early RRT.^[27,33]^ A multicenter high-quality RCT on this issue involved 620 patients with AKI of KDIGO stage 3.^[4]^ The early strategy started RRT within 6 hours of fulfilling KDIGO stage 3 AKI, while the delayed treatment strategy initiated upon fulfilling clinical criteria related to worsening AKI or complications. The primary outcome, mortality at 60 days, did not differ differently in the 2 groups: 48.5% (95% CI, 42.6–53.8) in the early-strategy group and 49.7% (95% CI, 43.8–55.0) in the delayed strategy group (*P* = 0.79). There was no difference in secondary endpoints including ventilator and vasoactive-free days through day 28, ICU stay, hospital stay, and 60-day dialysis. However, it is worth noting that only 61% of the patients in the delayed group received dialysis. A 12-center open-label pilot trial by Wald et al^[12]^ compared early (12 hours or less from eligibility) and standard RRT initiation in critically ill adults with volume replete severe AKI. Clinical outcomes were similar, all patients at 90 days following enrollment, with mortality 38% in the accelerated and 37% in the standard group. In another prospective randomized trial,^[23]^ earlier start of dialysis therapy before the onset of significant hyperkalemia, hypervolemia, or uremia did not result in improved survival (relative risk, 1.67; 95% CI, 0.88–3.17; *P* = 0.2) in patients with community-acquired AKI. They even reported that very early RRT delayed the recovery of kidney function in patients with sepsis. It was showed that RRT was associated with a higher mortality, a longer ICU, and hospital stay in comparison with conservative approach (volume, electrolyte, acid–base homeostasis, and specific drug management without dialysis) in patients with AKI.

However, these should be interpreted cautiously. There are already widely accepted indications for RRT in patients with AKI, which generally include refractory fluid overload; hyperkalemia (plasma potassium concentration >6.5 mEq/L) or rapidly rising potassium levels and/or ECG abnormalities; signs of uremia, such as pericarditis, bleeding, or encephalopathy; severe metabolic acidosis (pH < 7.15); certain alcohol and drug intoxications; and urine output less than 200 mL/12 h or anuria.^[6]^ The definition of early initiation is different from each other, which could explain the heterogeneity in these studies.^[34]^ By early initiation of RRT, clinicians may get better control of fluid and electrolyte status, removal of uremic toxins, and prevention of overt complications attributable to AKI before the patients becoming relatively resistant to therapy.^[7,12]^ But it may also expose them to the potential harms (e.g., hemodynamic instability, hemorrhage, thrombosis, and bacteremia). On the contrary, a delayed strategy may allow for spontaneous recovery of kidney function.^[4,12]^

### 4.1 Limitations

Our meta-analysis has several limitations. First, most studies were not comparable because definition of early was somewhat arbitrary and varies in the literatures (Table 2). A survey, distributed broadly to Canadian nephrologists and intensivists, showed that there was little agreement on what constitutes a trigger for initiation.^[35]^ In addition, the mortality was calculated according to the data of the researches with different follow-up time, which included 14, 30, 60, and 90 days. Second, the studies included were not conducted blindly. It was difficult to perform a double- or triple-blind method study. Third, there were differences in dose, mode, and intensity among these studies. However, there were balances between the 2 groups in each study. Fourth, the morbidity of each trail ranges differently. AKI is a multidisciplinary complication, and about 50% of AKI was caused by sepsis or septic shock. By subgroup analysis, we found similar results in postsurgery and septic patients.

**Table 2:**
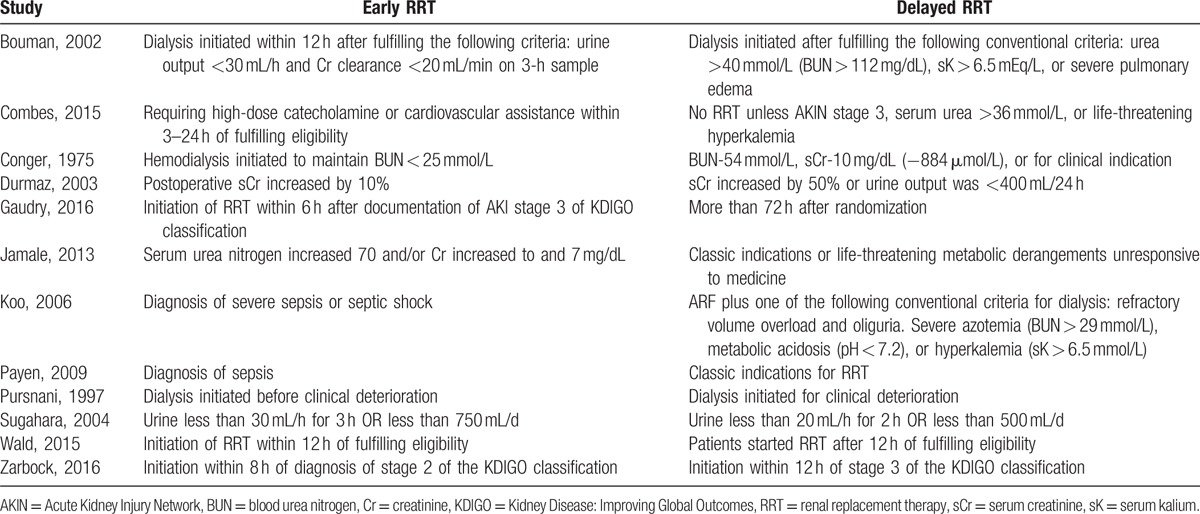
Definitions of early strategy and late strategy initiation of RRT.

## 5 Conclusion

In present systematic meta-analysis, early initiation of RRT showed no survival benefits in patients with AKI. In this difficult debate, we suggest that the decision to initiate RRT be made according to the specific condition of each patient with AKI. To achieve optimal timing of RRT, large multicenter randomized trials, with widely accepted and standardized definition of early initiation, are still needed.
